# Phase Entrainment of Brain Oscillations Causally Modulates Neural Responses to Intelligible Speech

**DOI:** 10.1016/j.cub.2017.11.071

**Published:** 2018-02-05

**Authors:** Benedikt Zoefel, Alan Archer-Boyd, Matthew H. Davis

**Affiliations:** 1 MRC Cognition and Brain Sciences Unit, University of Cambridge, 15 Chaucer Road, Cambridge CB2 7EF, UK

**Keywords:** phase, entrainment, speech, intelligibility, oscillation, transcranial alternating current stimulation, tACS, fMRI, BOLD, rhythm

## Abstract

Due to their periodic nature, neural oscillations might represent an optimal “tool” for the processing of rhythmic stimulus input [1, 2, 3]. Indeed, the alignment of neural oscillations to a rhythmic stimulus, often termed phase entrainment, has been repeatedly demonstrated [4, 5, 6, 7]. Phase entrainment is central to current theories of speech processing [8, 9, 10] and has been associated with successful speech comprehension [11, 12, 13, 14, 15, 16, 17]. However, typical manipulations that reduce speech intelligibility (e.g., addition of noise and time reversal [11, 12, 14, 16, 17]) could destroy critical acoustic cues for entrainment (such as “acoustic edges” [7]). Hence, the association between phase entrainment and speech intelligibility might only be “epiphenomenal”; i.e., both decline due to the same manipulation, without any causal link between the two [18]. Here, we use transcranial alternating current stimulation (tACS [19]) to manipulate the phase lag between neural oscillations and speech rhythm while measuring neural responses to intelligible and unintelligible vocoded stimuli with sparse fMRI. We found that this manipulation significantly modulates the BOLD response to intelligible speech in the superior temporal gyrus, and the strength of BOLD modulation is correlated with a phasic modulation of performance in a behavioral task. Importantly, these findings are absent for unintelligible speech and during sham stimulation; we thus demonstrate that phase entrainment has a specific, causal influence on neural responses to intelligible speech. Our results not only provide an important step toward understanding the neural foundation of human abilities at speech comprehension but also suggest new methods for enhancing speech perception that can be explored in the future.

## Highlights

• Phase lag between neural oscillations and speech rhythm was manipulated using tACS
• This manipulation modulated the BOLD response to speech in superior temporal gyrus
• tACS effects were observed for intelligible, but not unintelligible, speech
• Results show a causal role of neural entrainment during speech processing

## Teaser

Using simultaneous tACS-fMRI recordings, Zoefel et al. show that the alignment of neural oscillations to stimulus rhythm causally modulates neural responses to speech. The effect is specific for intelligible speech, supporting the notion that neural entrainment is a mechanism tailored to optimize speech processing.

## Results and Discussion

**Figure 1:**
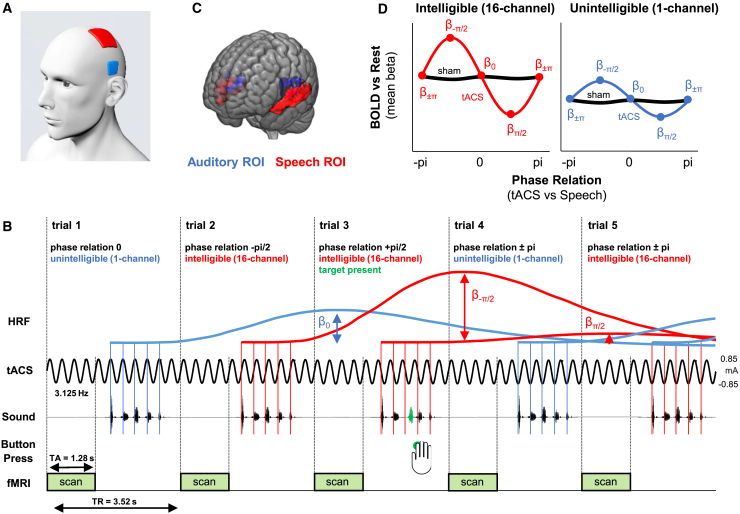
Experimental Paradigm, Analysis Methods, and Predictions (A) Electrode configuration. One 3 × 3 cm electrode (blue) was placed in position T7 of the 10-10 system overlying brain regions involved in speech perception (superior temporal gyrus [STG]), cf. (C). The other 5 × 7 cm electrode (red) was placed at position C3 of the 10-10 system. (B) During scanning runs with brain stimulation, tACS was applied continuously at 3.125 Hz and rhythmic speech stimuli with a matched syllable rate were presented at varying phase relations to the stimulating current. Speech stimuli were presented during the silent period in a sparse fMRI protocol such that the p-center [20] of all syllables fell at one of eight different phases of the applied current. Alignment of tACS and p-centers is indicated with vertical lines; four phase relations are shown for simplicity. As shown, we expected that the magnitude of the BOLD response would be modulated by tACS phase, which we can assess by fitting a hemodynamic response function (HRF) to BOLD responses for sentences presented at each phase relation (cf. D). Participants were given the task of detecting irregularities in the stimulus rhythm, introduced by advancing or delaying one of the five syllables (14% target trials; target syllable shown in green). (C) Two bilateral regions of interest (ROIs) were used for further analyses: a speech ROI (red), obtained by contrasting BOLD responses to intelligible (16-channel vocoded) and unintelligible (1-channel vocoded) speech, and an auditory ROI (blue), obtained by contrasting BOLD responses to unintelligible speech and a silent baseline. Speech ROI: p < 0.001, uncorrected, clusters >400 voxels; auditory ROI: p = 0.05, FWE-corrected, clusters >400 voxels. Note that different thresholds are used for visualization purposes. All ROI analyses were conducted using the same threshold (voxelwise p < 0.001; uncorrected; selecting clusters >400 voxels; corresponding to p < 0.05; cluster corrected). See Figure S1 for a more detailed depiction of these ROIs. (D) Predictions. We expected a sinusoidal modulation of the magnitude of the BOLD response to intelligible speech by the phase relation between tACS and stimulus rhythm (left). This sinusoidal modulation can be assessed using the parameter estimates (beta values) for the fitted HRF as depicted in (B). Effects of tACS on general auditory processing can also be assessed using BOLD responses to unintelligible speech (right). Irrespective of whether or not the tACS effect is specific to intelligible speech, we expect phase modulation to be absent for the sham condition (black).

To determine whether phase entrainment has a causal role in modulating neural responses, we used fMRI combined with transcranial alternating current stimulation (tACS) at 3.125 Hz over left lateral temporal regions (Figure 1A). Under the assumption that neural oscillations follow the imposed alternating current [19, 21, 22], we systematically varied the phase lag between tACS and rhythmically spoken speech (Figure 1B). Sentences were presented in silent periods during a sparse fMRI protocol and at variable delays such that the perceptual center (p-center [20]) of all syllables fell at one of eight different phases of the applied current (Figure 1B; see STAR Methods). We measured the consequences for neural responses to sentences consisting of five rhythmically spoken one-syllable words that were noise-vocoded to manipulate speech intelligibility (16-channel vocoded, i.e., intelligible, or 1-channel vocoded, i.e., unintelligible). Importantly, vocoded speech manipulates intelligibility while preserving critical elements of the speech rhythm (e.g., amplitude envelope). This allowed us to determine whether phase entrainment modulates neural responses for auditory processing per se (apparent for intelligible and unintelligible stimuli) or in a speech-specific fashion (specific to intelligible, 16-channel stimuli). Further evidence for speech specificity comes from using the high spatial resolution of fMRI to localize brain regions (Figure 1C; see also Figure S1) in which BOLD responses depend on the phase relationship between tACS and speech rhythm (predictions shown in Figure 1D). Effects of tACS were compared with a sham condition, in which stimulation was turned off after 6 s to produce sensations associated with the stimulation but without stimulating in the remaining ∼15 min of the block (e.g., [23]).

**Figure 2:**
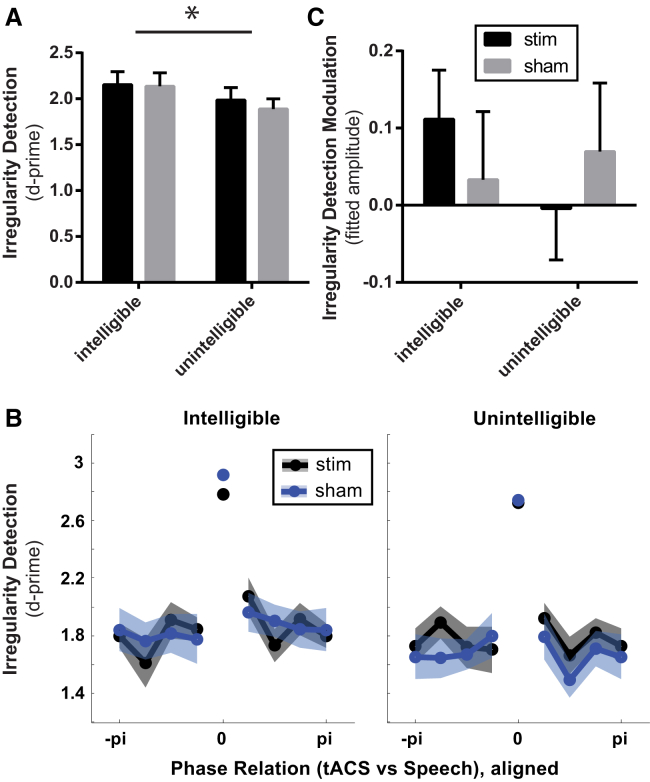
Behavioral Results (A) Performance in the behavioral task (measured as d-prime) in which participants had to detect irregularities in the speech rhythm. Note that performance is significantly better than chance (i.e., d-prime of 0) in all conditions. See Figures S2A and S2B for other behavioral measures (detection probability and false alarm probability). (B) Performance (d-prime) as a function of the phase relation between tACS and speech. Maximum performance was aligned at the center bin before averaging across participants. The shaded area shows SEM across participants (after removal of between-participant variation). See Figures S2C and S2D for corresponding plots using other behavioral measures. (C) A sine wave was fitted to the data shown in (B), separately for each participant. The center bin was excluded for this fit, and the phase of the sine wave was restricted so that its peak was aligned at the center bin (see STAR Methods for details). Shown is the average amplitude of this sine wave across participants, separately for each condition. SEM across participants is shown as error bars (after removal of between-participant variation). See Figures S2E and S2F for corresponding plots for other behavioral measures.

Our 17 participants were asked to detect an irregularity in the stimulus rhythm (green in Figure 1B; see STAR Methods). Behavioral analyses indicated that participants could reliably detect target trials in all conditions: d-prime (a signal detection measure of perceptual sensitivity, combining correct detections and false alarms [24]) was significantly above 0 (p < 0.0001; one-sample t test against 0), indicating substantially better than chance detection in all listening conditions. Performance was significantly better for intelligible than for unintelligible speech (Figure 2A; intelligible versus unintelligible: d-prime = 2.14 ± 0.52 versus 1.94 ± 0.46, mean ± SD; paired t test: t(16) = 2.37, p = 0.03; effect size, Cohen’s d: 0.42). This significant difference was mainly due to fewer false alarms in the intelligible (3.96% ± 2.82%) than in the unintelligible condition (Figure S2B; 7.51% ± 5.03%; t(16) = 3.22, p = 0.005; effect size: d = 0.83) and not due to a difference in detection probability (Figure S2A; intelligible versus unintelligible: 59.19% ± 14.61% versus 63.60% ± 16.31%; t(16) = 1.09, p = 0.29; effect size: d = 0.29). Performance did not differ between stimulation and sham conditions (Figure 2A; sham versus stimulation: d-prime = 2.07 ± 0.52 versus 2.01 ± 0.48; t(16) = 0.58, p = 0.57; effect size: d = 0.11). The behavioral task was not optimal for testing the effect of tACS phase on perception (because there were only 4 target trials per tACS phase bin in each condition) but intended to ensure that participants listened attentively to both intelligible and unintelligible stimuli. Perhaps because of this lack of power, when we analyzed the modulation of performance by the phase relation between tACS and speech (Figure 2B; see also Figures S2C and S2D; maximum performance was aligned at the center bin before averaging across participants), we were unable to find any statistically significant difference between conditions (three-way repeated-measures ANOVA with factors phase × stimulation × intelligibility, center bin excluded; all p values > 0.1). For each participant, we quantified the strength of behavioral modulation by extracting the amplitude of a sine wave fitted to the tACS-dependent changes in performance (i.e., to the data shown in Figure 2B, center bin excluded; see STAR Methods). Averaged amplitude values are shown in Figure 2C (see also Figures S2E and S2F). Again, no significant difference between conditions was revealed (two-way repeated-measures ANOVA with factors stimulation × intelligibility; all p values > 0.2).

Our fMRI analysis tested for a phase-specific modulation of the magnitude (but not the timing) of the hemodynamic BOLD response to our stimuli (Figure 1B). We anticipated that this BOLD modulation would show a sinusoidal dependence on the phase relation between tACS and speech (cf. function *f* shown in Figure 1D), which we assessed by fitting a sine wave to the BOLD response in each voxel for each of our conditions (relative to an unmodelled silent baseline) crossed with eight phase bins (see STAR Methods). Our electrode placement targeted the lateral temporal lobe, and we assessed tACS effects in auditory- and speech-responsive regions of interest (ROIs) that were defined using orthogonal contrasts. Our speech ROI (red in Figure 1C; see also Figure S1) was determined by contrasting the BOLD response to intelligible 16-channel speech (averaged over true and sham stimulation and all phase bins) with that to unintelligible 1-channel speech (similarly averaged) for the group of participants tested (cf. [25]). We also defined an auditory ROI (blue in Figure 1C; see also Figure S1) by contrasting the BOLD response to unintelligible 1-channel speech with a silent baseline [26]. Current flow during tACS is complex and determined by many different anatomical and experimental variables [19, 27, 28, 29, 30]. We therefore anticipated substantial individual differences in the voxels affected by stimulation as suggested by the aforementioned studies. Importantly, a strong effect that is present in different voxels for each participant or depends on local cortical orientation might be lost if data are averaged across participants on a voxel-by-voxel basis (as in conventional group analysis) or if data are averaged over multiple adjacent voxels (in conventional ROI analysis). We therefore determined, separately for each of our 17 participants and 4 conditions (i.e., the factorial crossing of stimulation/sham and 16-/1-channel speech), the 1% voxels with the strongest phasic modulation of the BOLD response in each of our ROIs (see STAR Methods). Note that, by using this selection procedure, we ensure that we will find non-zero phasic modulation of the BOLD response in the selected voxels; importantly, though, this procedure was applied identically for all conditions. Hence, our null hypothesis is still that there will be no difference in the strength of phasic modulation between conditions. In our first analysis, we therefore compared the *relative* strength of the tACS effect between conditions (e.g., stimulation versus sham).

**Figure 3:**
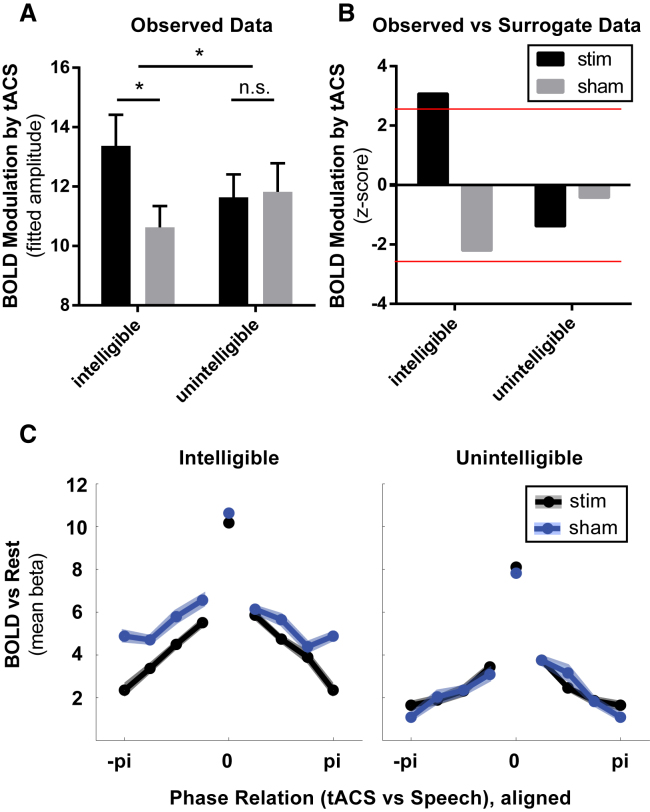
fMRI Results (A and B) For each voxel, condition, and participant, we determined the amplitude of a sine wave function *f* (Figure 1D), reflecting the magnitude of BOLD modulation due to differences in the phase relation between tACS and speech rhythm. (A) Mean sinusoidal amplitude of the 1% voxels with the strongest modulation, averaged across participants and voxels within the speech ROI (red in Figure 1C), shown separately for all conditions (error bars show SEM after between-participant variation has been removed as appropriate for repeated-measures analyses [31]). (B) Permutation tests confirmed that only the modulation effect in the stimulation/intelligible condition is significantly different from surrogate data obtained using a permutation procedure (red lines show the Bonferroni-corrected significance threshold of p = 0.05; two-tailed). Note that the amplitude pattern between conditions strongly resembles that observed for the modulation of behavior (Figure 2C). (C) BOLD response (i.e., beta estimates relative to silent baseline trials) as a function of the phase relation between tACS and speech, averaged across voxels (same voxels as used for A) and participants, and shown separately for the different conditions (SEM as described for A). For each voxel, condition, and participant, maximal responses were aligned at phase bin 0 before averaging to avoid phase cancellation effects. Note that this alignment is the only difference from the schematic illustration in Figure 1D. See Figure S3C for plots of single-participant data without phase alignment. The peak visible for the center bin is circular; it is shown separately from the other phase bins and excluded from analysis. The pi/−pi bin is plotted twice for visualization purposes. Note that, for some phase relations and only for intelligible speech, the BOLD response is suppressed by tACS stimulation.

Results obtained from this procedure are shown for the speech ROI in Figure 3A. A two-way repeated-measures ANOVA on the magnitude of phase modulation (factors: stimulation versus sham and intelligible versus unintelligible) yielded a significant interaction effect (F(1, 32) = 6.75, p = 0.014; effect size, partial eta-squared: $\eta_{partial}^{2}$ = 0.17), demonstrating that the stimulation-induced phase modulation of the BOLD response (i.e., the difference between stimulation and sham conditions) is significantly larger for intelligible than for unintelligible speech. Paired t tests confirmed that the observed difference between stimulation and sham conditions is significant in response to intelligible (t(16) = 3.73; p = 0.002; effect size: d = 0.69), but not for unintelligible, speech (t(16) = 0.22; p = 0.826; effect size: d = 0.05). No significant modulation of the BOLD response to unintelligible speech was found when data were analyzed in the auditory ROI (paired t test for stimulation versus sham: t(16) = 0.28; p = 0.785; effect size: d = 0.07). Moreover, as the broadband amplitude envelope (assumed to be critical for phase entrainment [8]) did not differ between intelligible and unintelligible speech (a property of noise vocoding [32], further discussed in [12]), the speech specificity of our tACS effect cannot be explained by trivial differences in the amplitude envelope of the stimulus.

Although our voxel selection procedure was applied equivalently to all four conditions and should therefore be unbiased, it remains necessary to assess which (if any) of the conditions shown in Figure 3A demonstrate reliable tACS modulation or whether there is any inadvertent bias created by differential BOLD responses to intelligible and unintelligible stimuli. We therefore compared the observed data in each condition with a surrogate distribution created by repeating the above analysis for 100 random assignments of single trials to different phase bins in each participant (including extracting the 1% voxels with strongest modulation for each permutation). The application of our voxel selection procedure in the surrogate distribution can provide us with a range of values for tACS-induced modulation of BOLD responses that would have been produced in a dataset in which no tACS effect is present (shown in Figure S3A). We found that only the modulation effect observed in the stimulation/intelligible condition differs significantly from the surrogate data (Figure 3B; effect size d = 0.76 for stimulation/intelligible condition; see Figure S3B for a voxel-by-voxel contrast with the corresponding surrogate data). That is, we only observed reliable tACS modulation of neural responses to intelligible speech. The absence of a neural effect of tACS on responses to unintelligible speech is in contrast to previous studies reporting a modulation of the detection of simple auditory stimuli by tACS phase at 4 Hz [33] and 10 Hz [34]. Although our study was designed to detect neural rather than behavioral effects of tACS, participants were nonetheless attending to the unintelligible stimuli: they responded with a high degree of accuracy in a detection task, and detection performance did not depend on the presence or absence of brain stimulation. The aforementioned studies reporting behavioral effects of tACS on auditory responses [33, 34] were shown with near-threshold stimuli—that is selecting stimuli for which the application of tACS should result in the most readily detectible shift of the psychometric function (i.e., making a given stimulus easier or harder to detect depending on tACS phase). Similarly, effects of perceptibility on neural responses in sensory cortex are largest for near-threshold stimuli [35, 36]. It is thus possible that our tACS protocol would have affected auditory processing, but we were unable to measure these effects (in behavior or neural responses), given that all stimuli were presented at a supra-threshold level. Critically, although relatively easy to understand, the *linguistic* properties of the 16-channel vocoded speech in our intelligible condition were relatively close to the threshold of intelligibility (i.e., 16-channel speech is degraded and not fully intelligible). This suggests that our tACS-induced modulation of neural responses to 16-channel speech could be accompanied by changes in intelligibility, a hypothesis that can be tested in future studies. Alternatively, it might be that the changes that we made to our stimulation protocol with respect to previous studies (e.g., electrode position and electrode size) were successful in targeting speech-responsive regions. It is known that the effect of tACS depends on neuronal orientation [37, 38], and it is therefore possible that stimulation parameters that are optimal for modulation of brain regions involved in processing sound in general (located in the lateral sulcus, e.g., primary auditory cortex [A1]) would differ from those that optimally modulate speech-processing regions (e.g., superior temporal gyrus [STG] [26]). Irrespective of the explanation for the speech specificity (due to behavioral parameters, i.e., perceptibility, or neural parameters, i.e., electrode configuration), we have shown that tACS leads to a specific modulation of brain regions involved in speech processing. This finding is inconsistent with the tACS effect being a downstream consequence of a more general modulation of auditory brain regions. Thus, our findings have important implications for models of speech processing in which phase entrainment serves as a critical underlying mechanism.

Nevertheless, some questions remain to be answered in follow-up studies. First, our stimuli consisted of speech recorded in time with a metronome, which made it straight-forward to align the p-center of each word with a specific phase of tACS (see STAR Methods). It is critical to develop strategies to transfer these findings to more natural speech stimuli. Although the latter has a less obvious rhythm than our stimuli, the spectrum of its amplitude fluctuations is nonetheless dominated by amplitude modulations in certain frequency ranges (∼1–8 Hz; e.g., [18, 39]), which, combined with listeners’ ability to track ongoing rhythmic fluctuations in acoustic input [4, 5, 6, 7, 8, 9], could provide a means for neural oscillations to entrain. These rhythmic amplitude fluctuations—reflected in the broadband speech envelope—could therefore be used as the current waveform for transcranial stimulation, potentially improving (or disrupting) neural entrainment when applied at an optimal (or non-optimal) lag relative to natural speech. However, this assumes that the speech envelope is neurally encoded and an important “cue” for entrainment—assumptions that are often made but rarely tested experimentally. Second, it is still debated whether the signal commonly measured as “entrainment” arises from intrinsic oscillatory dynamics or merely arises from a succession of evoked responses to a regularly occurring stimulus [40, 41]. Although there is evidence that entrainment can be “more” than regular evoked responses (for discussion, see, e.g., [42, 43]), we emphasize that the current study cannot answer this question: indeed, it is theoretically possible that the applied current interferes with these evoked responses, with the degree of interference depending on the phase relation between tACS and critical moments for speech processing. Further experiments in which tACS modulates speech processing after the current has been turned off would provide important evidence for modulation of phase entrainment. Previous work has shown that tACS effects on oscillatory amplitude can last several minutes (reviewed in [44]); however, it remains unclear whether the same applies for oscillatory phase (i.e., whether the aftereffects indeed reflect entrained oscillations; see [45] for a single negative finding).

Given the results presented so far, it is possible that modulation of phase entrainment by tACS leads to either enhancement or suppression of the BOLD response or both enhancement and suppression relative to non-stimulation (sham) conditions. To disentangle these alternatives, we determined the phase profile of the tACS effect by averaging the BOLD response to each phase bin over the 1% most strongly modulated voxels in each participant. The “best” (or “preferred”) phase for neural activity (i.e., the position of the peak response on the x axis in Figure 1D) differed across participants (Figures S3C and S3D). We therefore aligned the maximum BOLD response to phase (bin) 0 in each voxel before averaging over participants (cf. [46]). Results obtained from this analysis are shown in Figure 3C. A three-way repeated-measures ANOVA (main factors: phase relation [seven phase bins with the center bin excluded to avoid circularity], stimulation versus sham, and intelligible versus unintelligible) yielded a significant three-way interaction (F(6, 96) = 4.105; p = 0.0003; effect size: $\eta_{partial}^{2}$ = 0.23), confirming that, for some phase relations between tACS and speech rhythm, the BOLD response differs between stimulation and sham conditions, but this was only the case for intelligible speech. Interestingly, we only observed a tACS-induced suppression and no enhancement of the BOLD response to intelligible speech compared with the sham condition (Figure 3C). Note that this BOLD suppression might reflect a facilitation or a disruption of speech processing at particular phase relations. This reflects the non-monotonic, inverted-U-shaped relationship between speech intelligibility and BOLD responses that has been documented in previous fMRI studies [26, 47]; that is, a reduced BOLD response might be associated either with (1) decreased listening effort and improved intelligibility or (2) decreased neural engagement and, hence, reduced intelligibility (for further discussion of engagement/effort in spoken word recognition, see [48]); these alternatives can be disentangled in future studies. Consistent with the previous analysis, there was no tACS-induced suppression of responses to 1-channel, unintelligible speech.

**Figure 4:**
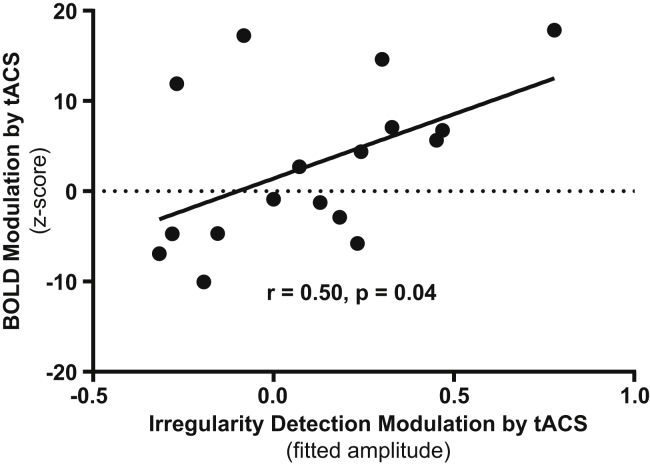
Correlation between Neural and Behavioral Results Correlation between tACS-induced modulations of BOLD responses and behavior (d-prime) by the phase relation between tACS and speech for the stimulation/intelligible condition. Individual z-transformed values of BOLD modulation were obtained by contrasting individual sine fit amplitudes (the average across participants is shown in Figure 3A) with a surrogate distribution that was calculated based on amplitude from a trial-based permutation analysis averaged across participants. For the calculation of tACS-dependent behavioral modulation, see STAR Methods. See Figure S4 for correlations between BOLD response and behavior in the stimulation/intelligible condition, using other measures of performance.

Even though the phase-dependent modulation of performance in our irregularity detection task was not significant in itself (reported above), it might nevertheless represent an adequate measure of how the observed BOLD modulation impacts perception. Indeed, in the stimulation/intelligible condition, the strength of tACS-induced BOLD modulation (Figure 3B) and the degree to which irregularity detection was modulated by the phase relation between tACS and speech (Figure 2C) were significantly correlated (Figure 4; see also Figure S4; detection probability: r = 0.51, p = 0.04; d-prime: r = 0.50, p = 0.04; no sig. correlation for false alarm probability as behavioral measure: r = 0.22, p = 0.39). No significant correlation between BOLD response and behavior was observed in any other condition (all p values > 0.1).

Our study provides evidence for a causal role of phase entrainment on neural responses to intelligible speech. These results are also in line with recent studies reporting that the perception of an isolated syllable depends on the phase of entrained oscillations [49] (but see [50]) and that entrained oscillations might influence the perception of subsequent speech [51]. Interestingly, at least in some studies, the latter effect seems to be speech specific [52], corroborating our results. However, the role of neural oscillations in mediating behavioral effects has previously been unclear. Whereas it is often proposed that phase entrainment of neural oscillations reflects a critical processing mechanism that is specifically adapted to the processing of speech sounds [8, 10], our work provides causal experimental evidence for this proposal. The speech specificity of our tACS effect demonstrates a crucial role of oscillatory phase entrainment for neural responses to speech that cannot be explained by general auditory mechanisms. We also demonstrate that, if stimulation protocol and experimental parameters are designed carefully, tACS is a promising technique for manipulating neural activity, in contrast to criticisms raised elsewhere [53, 54]. Thus, our results not only provide an important step toward understanding human abilities at speech comprehension but also suggest new methods for enhancing speech perception that will be explored in future studies.

## STAR★Methods

### Key Resources Table

**Table:** 

REAGENT or RESOURCE	SOURCE	IDENTIFIER
**Deposited Data**		

Raw and analyzed data	This paper	https://doi.org/10.17863/CAM.16677

**Software and Algorithms**		

Custom-built MATLAB code	This paper	https://doi.org/10.17863/CAM.16677
MATLAB 2014a	The MathWorks	http://www.mathworks.com
Statistical Parametric Mapping 12	[55]	http://www.fil.ion.ucl.ac.uk/spm/software/spm12/
Automatic Analysis (aa)	[56]	http://automaticanalysis.org/
Circular Statistics Toolbox	[57]	https://philippberens.wordpress.com/code/circstats/
Praat	[58]	http://www.praat.org/

**Other**		

DC-Stimulator MR	Neuroconn	http://www.neurocaregroup.com/dc_stimulator_mr.html
Quad-Capture sound card	Roland Ltd	https://www.roland.com/uk/products/quad-capture/
Sensimetrics insert headphones	Sensimetrics Corporation	http://www.sens.com/products/model-s14/

### Contact for Reagent and Resource Sharing

Further information and requests for resources and reagents should be directed to and will be fulfilled by the Lead Contact, Benedikt Zoefel (benedikt.zoefel@mrc-cbu.cam.ac.uk).

### Experimental Model and Subject Details

Twenty-two participants were tested after giving informed consent in a procedure approved by the Cambridge Psychology Research Ethics Committee. Three participants did not finish the experiment as they were not comfortable in the fMRI scanner, one participant reported falling asleep repeatedly, and the data from one participant could not be analyzed due to technical problems, leaving seventeen participants (10 female) in the study for further analyses. All were native English speakers, aged 23-52 years (33 ± 8 years, mean ± SD) and had no history of hearing impairment, neurological disease, or any other exclusion criteria for fMRI or tACS based on self-report.

### Method Details

#### Stimuli

Speech is not a perfectly rhythmic stimulus so that a sinusoidal current applied with tACS might not perfectly align with the speech rhythm. In principle, the current waveform used for tACS is arbitrary (i.e., not necessarily sinusoidal) and might be adapted to certain characteristics of the speech signal that are associated with its rhythmicity (such as its envelope). However, to avoid uncertainties concerning the auditory signals that convey speech rhythm we decided to construct rhythmically spoken five-syllable sentences that are perceptually aligned to a metronome beat (sentence structure: “pick” <number> <color> <animal> <direction>; example: “pick one red frog up”). These sentences were recorded by a male native English speaker (author MHD) at 2 Hz spoken in time to a metronome recorded on a separate channel. After recording, sentences were up-sampled to 3.125 Hz by time-compression using the pitch-synchronous overlap and add (PSOLA) algorithm implemented in the Praat software package [58]. Recording at a slower rate increased the clarity of the recordings, and improved the ability of the speaker to produce the spoken words separately (such that words could be combined between sentences) compared to recordings made at a faster rate.

In this way, we were able to obtain a large set of sentences that are perceptually rhythmic and with “Perceptual centers,” or “p-centers” [20, 59] that are aligned with a metronome beat. This procedure results in at least two advantages relative to using more natural sentences: First, we can apply sinusoidal tACS and align it to regular rhythmic events in speech (in perceptual terms, i.e., p-centers), thereby reducing the complexity of the experimental protocol. Second, by increasing the perceptual rhythm of the stimulus, we also aimed to enhance the entrainment of neural oscillations to the stimulus rhythm and, consequently, the modulation of the latter by tACS. The metronome beat was only used during stimulus construction and was not audible to participants.

Noise-vocoding is a commonly used technique for the systematic manipulation and degradation of speech stimuli [32]. We used this method to construct two degraded speech conditions derived from the (up-sampled) rhythmic sentences that were recorded: One condition used sentences that are clearly identifiable as speech (16-channel vocoded sentences) and another used physically similar stimuli that (due to the lack of spectral detail) sound like an amplitude modulated broadband noise that although it resembles speech cannot be recognized in isolation (1-channel vocoded). Importantly, noise-vocoding does not alter the rhythmic fluctuations in sound amplitude of the stimulus that are commonly assumed to be important for phase entrainment [8]. Thus, non-specific acoustic differences between the two stimulus two conditions are unlikely to be responsible for differences in neural responses.

#### Experimental Design

In this study, we manipulated phase entrainment to rhythmic speech/noise (in the 16-channel, intelligible condition) and noise sounds (in the 1-channel, unintelligible condition) and measured the consequences for the BOLD response to these stimuli in brain regions associated with speech and auditory processing, respectively. For this purpose, we applied tACS with the assumption that neural oscillations would follow the imposed electrical current [19, 21, 22]. Thus, we were able to control the phase relation between neural oscillations (reflected by the applied current) and auditory input rhythm. 8 phase relations (between ± π and 3/4 π, in steps of 1/4 π) between tACS and auditory stimuli were tested; in practice, tACS was applied continuously and the presentation of the rhythmic sounds (i.e., the p-centers of all syllables, see above) was timed to be aligned with a certain tACS phase (Figure 1B).

The experiment consisted of 4 runs of 256 trials each (128 trials per run for each of the 2 degraded speech conditions). In run 1 and 4, sham stimulation (sham condition) was applied by ramping the current up and down (following a Hanning window of 6 s length) immediately at the start of the scanning run. This created the usual sensations associated with tACS but without ongoing tACS during the remainder of the scanning run (e.g., [23]). tACS was applied continuously in run 2 and 3 (stimulation condition) and stimulation was turned off between scanning runs. In total, each run was approx. 15 min long. Participants were given time to rest in the scanner between runs when requested.

Each trial (Figure 1B) was 3.52 s long (based on the MRI scanner repetition time, TR) and started with the acquisition of a single MRI volume (TA = 1.28 s). During the remainder of the trial (2.24 s), the scanner was silent in order to avoid interfering effects of scanner noise on the presented auditory stimuli [60]. The scan was followed by a silent period corresponding to one cycle of the stimulus rhythm (1/3.125Hz = 320 ms) plus an interval that depended on the phase relation between tACS and stimulus in the respective trial (between 0 and 280 ms, in steps of 40 ms; see Figure 1B). After the silent period, the auditory stimulus (i.e., a single five-syllable sentence of 16-channel or 1-channel vocoded speech) was presented, with a duration of 1.6 s. After the stimulus, there was another silent period until the beginning of the next scan/trial (between 40 and 320 ms, depending on the phase relation of the respective trial). In 32 of the 256 trials in each run, no sound was played in order to enable a comparison of neural activity elicited by intelligible/unintelligible speech with a silent baseline (see below). Stimuli (16- or 1-channel vocoded) and phase relation (8 possibilities) was chosen (pseudo-)randomly for each trial, and counterbalanced between runs, resulting in (256-32)/8/2 = 14 trials per phase and condition in each run. Identical stimulus presentation conditions were included in sham scanning runs. Together, our experimental protocol resulted in a 2 × 2 x 8 factorial design with factors intelligibility (16-channel, intelligible versus 1-channel, unintelligible), stimulation (stimulation versus sham) and phase lag (8 possible lags as described above). Our analysis focused on the amplitude of phasic modulation of the BOLD response for four conditions (intelligible/stimulation, intelligible/sham, unintelligible/stimulation, unintelligible/sham).

In order to ensure participants remained attentive throughout the experiment, one of the five (but excluding first and last) syllables in the stimulus rhythm was shifted in time (±68 ms) on a small proportion of trials (14%) divided equally between intelligible and unintelligible conditions and phase relations (shown in green in Figure 1B). Participants were given the task of detecting these shifts and indicate their detection with a button press of the right index finger. Feedback on the level of correct performance was given verbally after each scanning run.

#### Electrical Stimulation

Current was administered using an MRI-compatible, battery-driven stimulator (DC-Stimulator MR, Neuroconn, Ilmenau, Germany). The stimulator was driven remotely by the output of one channel of a high-quality sound card (Roland Quad-Capture, Swansea, UK); another output channel was used to transmit monophonic, diotic auditory stimuli to the participants’ headphones in the scanner (Sensimetrics insert headphones, Sensimetrics Corporation, Malden, MA, USA, model S14), ensuring consistent synchronization between applied current and speech stimuli.

Current flow during transcranial current stimulation is complex [19, 27, 28, 30] and requires further investigation, in particular for the stimulation of the auditory system [61, 62]. Based on promising previous studies (e.g., [33, 34]), we decided to place one electrode in position T7 of the 10-10 system (Figure 1A), overlying brain regions involved in speech perception (e.g., Superior Temporal Gyrus, STG; *cf.* Figure 1C). The other electrode was placed at position C3 of the 10-10 system. Note that, at a given moment in time, the alternating current below the two electrodes is expected to show phase opposition [21] which might lead to oscillations entrained to opposite phases and unclear effects on neural activity. It has been suggested that current density can be increased for one electrode by reducing its relative size while keeping current intensity constant [63]. This approach might increase the relative impact on oscillatory entrainment for brain regions beneath the smaller (as compared to the larger) electrode. We therefore reduced the size of the electrode over T7 (3 × 3 cm) as compared to that over C3 (5 × 7 cm). However, note that the cited study based its claims on effects on the excitability of the motor system and is not undisputed [64]; indeed, some authors have cautioned against over-emphasizing the effects of one electrode while ignoring potential effects of the other [62, 65, 66]. Some studies also reported that current flow might not be maximal below but rather between electrodes [67, 68], although other work suggested that this might only be the case for specific stimulation parameters and/or assumptions underlying models of current flow [27]. Together, these factors necessitate testing alternative electrode positions and stimulation parameters in the future and underline the benefit of combining tACS with imaging methods such as fMRI so that effects of tACS on neural activity can be characterized with high spatial resolution. Electrodes were kept in place with adhesive, conductive ten20 paste (Weaver and Company, Aurora, CO, USA). Current intensity was set to 1.7 mA (peak-to-peak). After each run, participants were asked to rate the perceived side effects of the stimulation between 0 (no side effects) and 10 (very strong side effects). On average, stimulation runs were rated as giving numerically higher side effects (1.49 ± 1.57, mean ± SD) than sham runs (1.21 ± 1.37), but the two stimulation conditions did not differ significantly (t(16) = 0.99, p = 0.33; paired t test).

#### fMRI Data Acquisition and Pre-processing

MRI data were acquired on a 3-Tesla Siemens Prisma scanner using a 64-channel head coil. A T1-weighted structural scan was acquired for each subject using a three-dimensional MPRAGE sequence (TR: 2250 ms, TE: 3.02 ms, flip angle: 9 deg, spatial resolution: 1x1x1 mm, field-of-view: 192x256x256 mm). We used sparse imaging [60] to acquire fMRI data. For each participant and scanning run, 260 echo planar imaging (EPI) volumes (after exclusion of initial dummy scans) each scan comprising 38 slices of 3 mm thickness acquired using a continuous, descending acquisition sequence with multi-band acceleration (TR: 3520 ms, TA: 1280 ms, TE: 30 ms, flip angle: 87 deg, matrix size: 38x64x64, in plane resolution: 3x3x3 mm, inter-slice gap 25%, acceleration factor: 2x). TR and TA were chosen based on prior observations that, although tACS does not seem to produce artifacts in the MRI signal [67], this might depend on TR and TA being an integer multiple of the period of stimulation frequency (i.e., a multiple of 320 ms such that the net stimulation current during the period of one MRI acquisition is zero and all scans begin at the same tACS phase).

fMRI data were pre-processed using SPM12 (http://www.fil.ion.ucl.ac.uk/spm) applying automatic analysis (aa) pipelines [56]. Pre-processing included the following steps for each participant: (1) re-alignment of each EPI volume to the first scan of the first run combined with correction for geometric distortions [69], (2) co-registration of the structural image to the mean EPI, (3) normalization of the structural image to a standard template, (4) application of the normalization parameters to all EPI volumes including re-sampling to a voxel size of 2x2x2 mm. Finally, (5) spatial smoothing was applied using a Gaussian kernel with a full-width at half maximum (FWHM) of 8 mm. This smoothed data was used for the analysis of average BOLD responses combined over phases and stimulation/sham conditions (used to generate regions of interest, ROIs, see below). Analyses to determine effects of tACS (see below) were run on unsmoothed fMRI data as the impact of the applied current on membrane depolarization depends on the precise orientation of the cortical surface with respect to the electric field [37, 38]. Since cortical orientation might differ markedly between adjacent voxels – for instance inside the superior temporal sulcus adjacent voxels might come from cortical surfaces with opposite orientations with respect to the electric field – conventional spatial smoothing could mix tACS effects originating from cortical patches with very different preferred phases and thereby obscure effects of tACS phase on fMRI responses.

Analysis of each participant’s pre-processed fMRI data was conducted using a general linear model (GLM) in which the four scanning runs (two stimulation runs and two sham runs) were modeled separately in a single design matrix in which there were separate event-related regressors for each phase relation and stimulus (i.e., 2 × 8 = 16 conditions for each scanning run). Six realignment parameters were included in each run to account for movement-related effects and four regressors were used to remove the mean signal from each of the runs. An AR(1) correction for serial autocorrelation was applied and a high-pass filter with a cutoff of 128 s included to eliminate low-frequency signal confounds such as scanner drift. These single participant models were fitted using a least-mean-squares method to each individual’s data, and parameter estimates (i.e., beta values) were obtained for all voxels for each of the 17 participants, each of the 2 scanning runs (sham and stimulation conditions), each of the 8 phase relation, and each of the 2 stimulus types (intelligible and unintelligible sentences). These beta values were used for further data analyses as described below.

### Quantification and Statistical Analysis

We assumed that the BOLD response measured by fMRI is a good proxy for neural activity [70] and hypothesized that the BOLD response will be modulated by the phase relation between tACS and stimulus rhythm. If phase entrainment were indeed critical for speech (or auditory) processing, there should be one or more phase relation(s) between tACS (i.e., imposed neural oscillations) and speech rhythm that significantly modulates neural responses (a “preferred” phase). Conversely, there should be other phase relations (close to the preferred phase) that produce a lesser modulation and other, more distant, phase relations that produce no modulation of neural responses or even an opposite modulation (i.e., if the preferred phase enhances the BOLD response relative to sham stimulation, more distant phases could produce suppression of the BOLD response). Given the existence of some preferred phase, we therefore predicted a modulation of the BOLD response that will follow a sinusoidal pattern as a function of the phase relation between speech and tACS. The magnitude of this sinusoidal modulation was assessed by using parameter estimates (beta values) from the single-subject statistical model described above and shown schematically in Figures 1B and 1D. These beta-values can then be analyzed as a function of the phase relation between tACS and stimulus rhythm in individual voxels (examples of this function *f,* assuming stimulation can both enhance and suppress the BOLD response, are shown in Figure 1D). The amplitude of a sine wave fitted to *f* reflects how strongly the BOLD response is modulated by the phase relation between tACS and stimulus rhythm. Note that an overall change in neural activity that is independent of phase bin (e.g., in speech-responsive areas, such as STG, neural activity might be stronger in response to speech than to noise) would only result in a “baseline shift” of *f* (e.g., compare intelligible and unintelligible conditions in Figures 1D and 3C) and the fitted sine wave but not affect the latter’s amplitude. We extracted the amplitude of the fitted sine wave for each voxel, condition, and participant, in order to quantify our tACS effects. For each phase bin, and separately for each voxel, condition, and participant, beta values were averaged across both runs before the sine wave was fitted; in this way, only effects with a preferred tACS phase that is consistent across runs (for a given voxel) would show a reliable phase modulation effect on neural responses. This procedure improved the signal-to-noise ratio for our hypothesized effect as this phase consistency would only be expected for stimulation (but not sham) runs.

We anticipated that tACS-induced modulation of BOLD responses might only be present at specific locations in the brain: Indeed, if phase entrainment played an important role in speech comprehension [9, 11, 12, 13, 15], we would expect the tACS effect to be maximal in regions that were specifically engaged in processing speech sounds. On the other hand, phase entrainment can be observed in response to simple acoustic stimuli, such as regularly repeating pure tones [4], which suggests the possibility of effects of tACS on areas processing auditory input in general. We therefore defined two functional ROIs (Figures 1C and S1) to be used for statistical analysis: (1) A speech ROI assessed from a group analysis using a paired t test that assessed the differential BOLD response to 16-channel, intelligible speech (averaged across runs, stimulation conditions and phase relations) with that to the 1-channel, unintelligible speech (averaged as before). This contrast reveals a speech-responsive region covering the bilateral STG and middle temporal gyrus (MTG) [25]; this ROI is shown in red in Figure 1C. (2) An auditory ROI obtained by computing in a group analysis the contrast between BOLD responses to the 1-channel, unintelligible speech (averaged over runs, stimulation conditions and phase relations) compared to an (unmodelled) silent baseline. This contrast reveals an area mostly restricted to Heschl’s gyrus (i.e., primary auditory cortex [26]); this ROI is shown in blue in Figure 1C. For all analyses, we adopted a significance threshold of voxelwise p < 0.001, uncorrected, and selected clusters > 400 voxels (all of which exceed p < 0.05, cluster corrected) for both ROIs.

Current flow during tACS is determined by different variables, including individual anatomy [19, 29, 30]. We therefore anticipated substantial individual differences in the voxels affected by stimulation and adapted our analysis procedures accordingly: We extracted for each condition and participant the 1% voxels with the strongest BOLD modulation in each of our two ROIs (speech or auditory), i.e., the (∼40) voxels with the highest amplitudes of the sine wave fitted to the BOLD response over phase bins. For each participant, the same ROI defined based on a group analysis was used. Amplitude values were then averaged over these selected voxels for each participant, and values from each participant for each stimulation condition and stimulus type (intelligible/unintelligible) were compared between conditions (using ANOVAs and paired t tests; see main text). Note that the null hypothesis of no differences between conditions in terms of the magnitude of sinusoidal modulation was still valid as this voxel selection and averaging procedure was applied identically for all conditions. Note also that our approach (fitting sine waves to *f* and extracting the highest amplitude values) will inevitably yield mean amplitude values larger than 0 for each of the conditions. It is therefore difficult to determine using parametric statistics whether the observed amplitude in a given voxel or condition is reliably greater than would be expected by chance. We therefore constructed a surrogate distribution by randomly assigning single trials to different phase bins in each participant and scanning run and repeating the analysis described above 100 times. For each of the 100 permutations, and separately for each condition, we extracted the mean amplitude value across voxels (again, for the 1% voxels showing the largest sinusoidal modulation) for each of the 17 participants; we were thus able to construct a distribution of amplitude values that would be observed by chance. For each condition, we separately transformed the observed sinusoidal amplitude values into statistical (z-)values by comparison with the surrogate distribution: z=a−μ/σ, where z is the z-transformed observed data, a is the observed data (i.e., amplitude averaged across voxels and participants), and μ and σ are mean and standard deviation of the surrogate distribution, respectively. The effect observed in a given condition was considered reliable if the z-value exceeded a critical value (z = ± 2.5, corresponding to the Bonferroni-corrected significance threshold of p = 0.05, two-tailed for four conditions).

Target trials (i.e., trials in which one of the five syllables was shifted toward another) were included in the analysis, as target occurrence was unpredictable (and thus effects on phase entrainment were unlikely) and four out of five syllables were still aligned with the intended tACS phase (such that BOLD modulation would still be expected for target-present trials). A re-analysis of the data with target trials excluded did not change any of the results reported in the main text.

In addition to potential BOLD effects, we also analyzed whether certain measures of performance in our behavioral task (probability of a target detection, i.e., “hit,” probability of false alarm and d-prime, the z-transformed difference between the two measures [24]) are modulated by the phase relation between tACS and speech. We therefore calculated performance as a function of this phase relation and quantified the strength of behavioral modulation by extracting the amplitude of a fitted sine wave, similar to the analysis of BOLD modulation described above.

However, as the behavioral task was mainly included to ensure participants remained alert, this analysis was based on only 4 target trials (and 28 non-target trials) per phase bin and condition. This small number of trials increased unexplained variance (“noise”) in the data and the likelihood of a poorly-fitting sinusoidal modulation. We therefore restricted the sine fit based on the following procedure. First, maximum performance (i.e., highest detection probability, highest false alarm probability, or highest d-prime) was assigned to the center bin (0 phase) for each participant (see, e.g., [46]). A sinusoidal modulation of performance should still be apparent, even without data from the center bin (i.e., the peak of the function). For each participant, we therefore fitted a sine wave to this function (i.e., aligned behavioral data, excluding center bin; the average across participants is shown in Figures 2B and S2C and S2D) and constrained its phase so that the peak of the sine wave was aligned with the center bin. In this way, only a sinusoidal modulation of performance (independent of “preferred” tACS phase) would be expected to result in large amplitude values for the fitted sine wave; these extracted amplitude values were used for the correlation analyses depicted in Figures 4 and S4, and their average across participants is shown in Figures 2C (d-prime) and S2E and S2F (detection probability, false alarm probability). Note that amplitudes can take on negative values: In this case, the sine wave is flipped, i.e., its trough is aligned with the center bin. This also means that the null hypothesis is well-defined as an amplitude value of 0 and the construction of a surrogate distribution is not necessary.

MATLAB 2014a (The MathWorks) was used for all analyses described, along with SPM version 12 [55] and the toolbox for circular statistics [57] where appropriate.

### Data and Software Availability

Data and custom-built MATLAB scripts (including stimulus construction) are available at https://doi.org/10.17863/CAM.16677.

## Supplemental Information

Document S1. Figures S1–S4

Document S2. Article plus Supplemental Information
