# Supplementary material for: Phase Entrainment of Brain Oscillations Causally Modulates Neural Responses to Intelligible Speech
Source: Curr Biol. 2018 Feb 5;28(3):401–408.e5. doi: 10.1016/j.cub.2017.11.071 (PMC5807089; doi:10.1016/j.cub.2017.11.071)
Supplement: Document S1. Figures S1–S4 [file mmc1.pdf]

Current Biology, Volume *28*

Supplemental Information

Phase Entrainment of Brain Oscillations Causally Modulates Neural Responses to Intelligible Speech

Benedikt Zoefel, Alan Archer-Boyd, and Matthew H. Davis

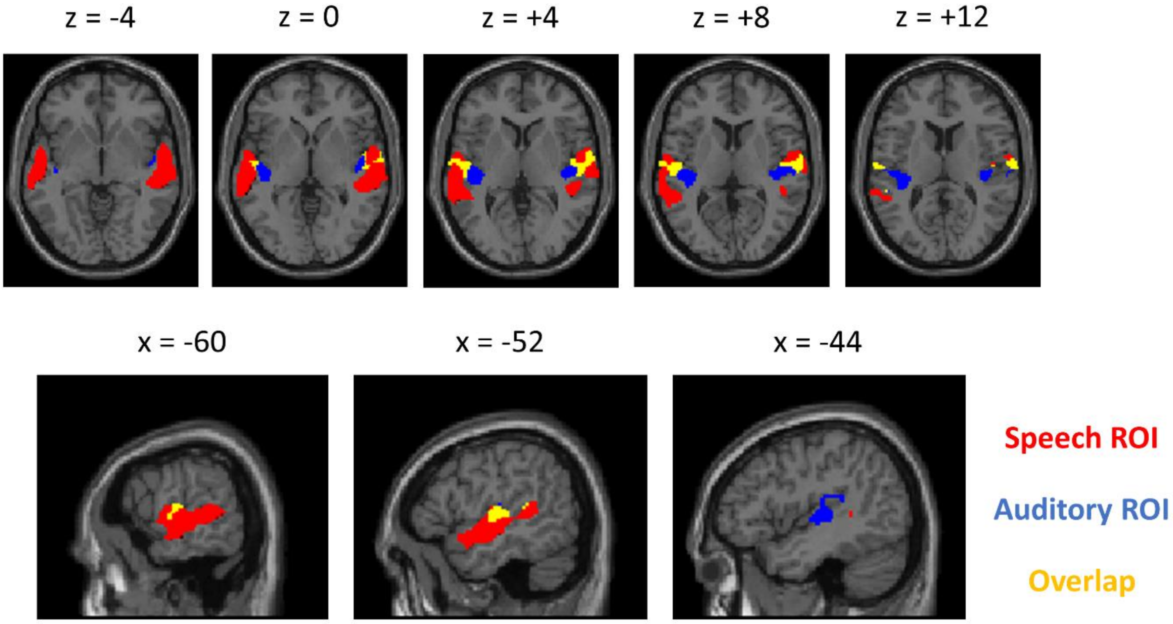

Figure S1. Regions of Interest (ROI). Related to Figure 1C. Axial (top) and sagittal (bottom) representation of the two ROIs defined in this study overlaid on single slices of a canonical brain image with Z and X coordinates as shown (speech ROI: red; auditory ROI: blue; overlap of the two ROIs: yellow). For all other conventions, see legend of Figure 1C.

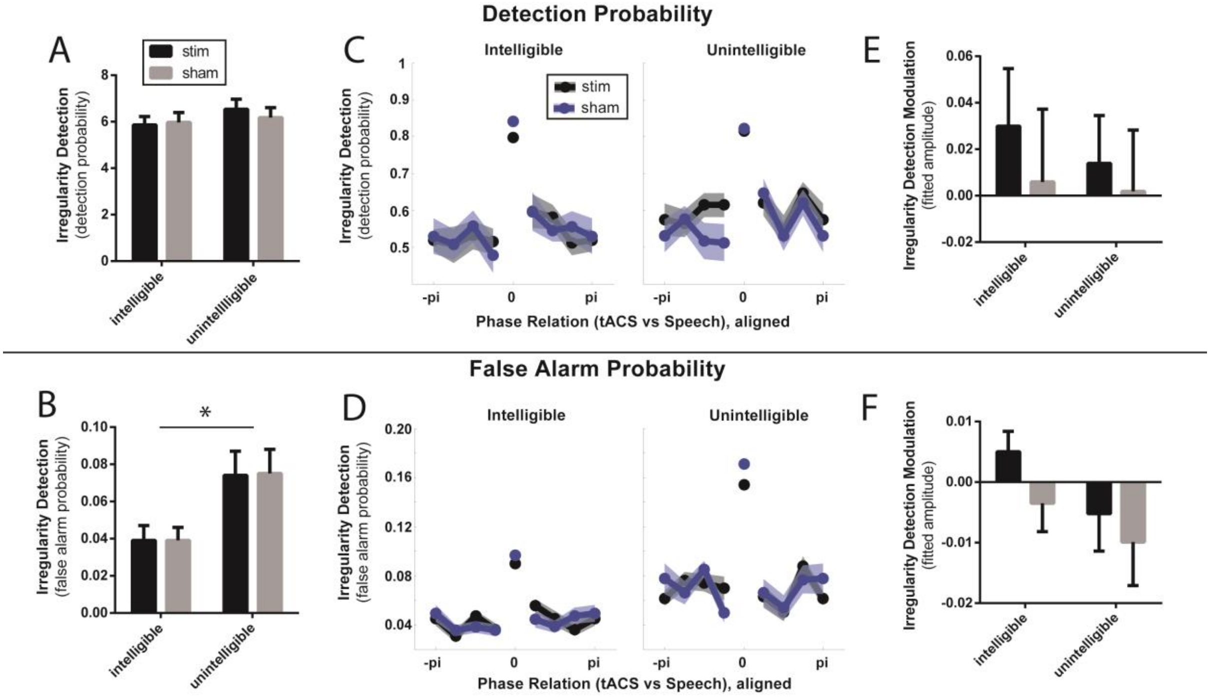

Figure S2. Supplemental Behavioural Results. Related to Figure 2. **A,B**. Performance in the irregularity detection task using other measures of performance (detection probability, A; false alarm probability, B). Note that the significant difference in d-prime between intelligible and unintelligible conditions, shown in Figure 2A, is mainly due to a significantly lower probability of false alarms in the intelligible conditions. **C,D**. Behavioural performance as a function of the phase relation between tACS and speech, for detection probability (C) and false alarm probability (D) as dependent measures, plotted as in Figure 2B. **E,F**. Average amplitude of sine waves fitted to the data shown in C,D (excluding centre bin; see also STAR Methods). Note that amplitude values do not differ significantly between conditions (see main text for statistics). However, not only is the mean amplitude across participants highest in the stimulation/intelligible condition for all measures of performance (including d-prime shown in Figure 2C), it is also significantly larger than 0 (the null hypothesis, see STAR Methods) in this condition (only) when analysed for false alarm probability (panel F; $t(16) = 2.54$, $p = 0.02$; effect size $d = 0.61$). See Figure S4 for the correlation between these amplitude values and those obtained for the BOLD modulation. For other details, see legend of Figure 2.

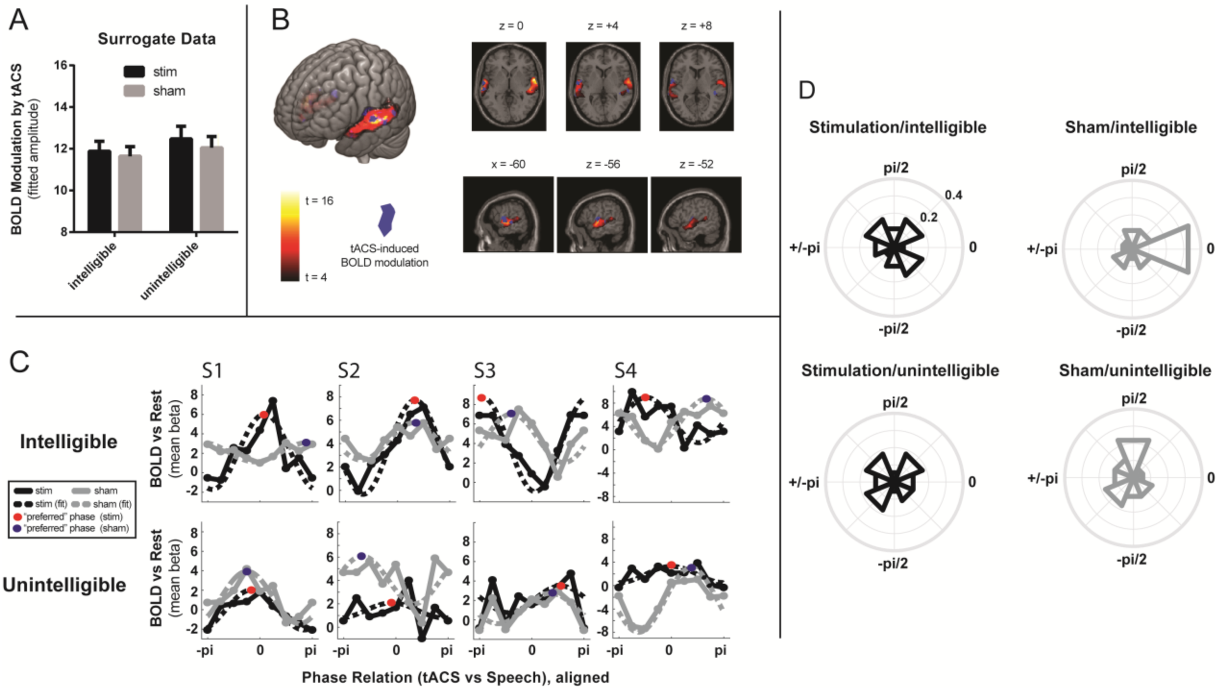

<u>Figure S3</u>. Supplemental fMRI Results. Related to Figure 3. **A**. TACS effects were quantified by fitting a sine wave to the tACS-induced BOLD modulation in each voxel, and extracting the 1% voxels with the strongest BOLD modulation (reflected in the amplitude of the fitted sine wave) for each condition. As the mean amplitude in these voxels is necessarily larger than 0, this procedure makes it difficult to judge whether an effect observed in a given condition (e.g., as shown in Figure 3A) is reliable i.e. statistically significant. We therefore constructed surrogate distributions (by shuffling the assignment of trials to phase bins; see also STAR Methods) – these distributions can show us what our analytical approach (e.g., the "voxel selection" procedure) would produce if applied to a dataset in which no effect can be expected. Shown here are the characteristics of these distributions: Mean extracted amplitudes across 100 permutations are shown as bars, and the standard errors of mean (SEM) across permutations as error bars. Mean and variance across permutations can then be compared with the observed data (Figure 3A), resulting in a z-score for each condition (Figure 3B) that can readily be interpreted statistically. Note that amplitudes extracted for the surrogate distribution do not significantly differ between conditions (all p's > 0.05; obtained by contrasting the mean amplitude across permutations in one condition with the range of amplitudes observed across permutations in another, for all possible combinations of conditions), confirming that our voxel selection procedure did not bias our analysis in favour of any specific condition. **B**. In order to take into account individual differences in tACS current flow and neural effects of stimulation (see STAR Methods and Results), the results presented in Figure 3 are based on analyses using data from different voxels for different participants. This makes it difficult to determine where tACS effects are – on average – strongest. For each voxel in the speech ROI, we therefore averaged the amplitude of the fitted sine wave (see STAR Methods) across participants and compared the results with the mean and standard deviation of surrogate data in the same voxel, resulting in a z-score for each voxel. This figure displays z-scores in blue on a rendered canonical brain (left panel), on axial slices (top right panels) and on sagittal slices (bottom right panels). We show results only for the intelligible/stimulation condition since this is the only condition for which a reliable BOLD modulation was observed (see Results). Note that, for this analysis, individual differences in current flow are not taken into account, strongly reducing effect size. For clarity, an uncorrected significance threshold of $p = 0.05$ (two-tailed; corresponding to a threshold of $z = \pm 1.96$) has been applied (voxels above that threshold are shown in blue). To assist in visualising, we also overlay a t-map for the contrast of intelligible vs unintelligible speech in bilateral STG/MTG, thresholded at $p < 0.001$, uncorrected (both clusters shown exceed $p < 0.05$, FWE cluster corrected) – the contrast that was used to define the speech ROI (see Figure 1C). Nonetheless, we emphasize that the aim of this analysis is not to demonstrate statistical significance (as this has already been done in Figure 3), but rather to characterize the approximate location of the significant findings reported in the main text. Several clusters are observed with a (relatively) strong tACS-induced BOLD modulation. However, since the strength of this modulation is most likely determined by an interaction between electric field and neural activity [S1,S2], and the detailed properties are currently unknown. It is difficult to make strong claims concerning the exact anatomical location of these BOLD-tACS effects. **C,D**. "Preferred" phase relations between tACS and speech (i.e. the phase relation producing the largest BOLD response) differ across participants. Shown in C is the BOLD response as a function of the phase relation between tACS and speech for four exemplary participants (S1-4), averaged across ~40 selected fMRI voxels (continuous lines). The distribution of "preferred" phase in these selected voxels was significantly biased ($p < 0.05$; Rayleigh's test) towards one phase for at least 15 out of 17 participants in each condition; it was thus possible to average tACS-dependent BOLD responses across these voxels without strong phase cancellation effects. Dotted lines show sine waves fitted to the averaged BOLD signals in each condition; red and blue dots show "preferred" phases in stimulation and sham condition, respectively, determined as the phase of the sine fit that corresponds to the largest BOLD response. Note that data was averaged across selected voxels only for the purpose of illustrating individual "preferred" phases; this was not necessary for the analyses described in the main text as tACS-dependent BOLD modulation was analysed at the level of single voxels. Shown in D is the distribution of "preferred" phases (i.e. those shown using red and blue dots in C) across all (N=17) participants, separately for each condition. None of the phase distributions shown is significantly biased towards one phase (all p's > 0.12; Rayleigh's test).

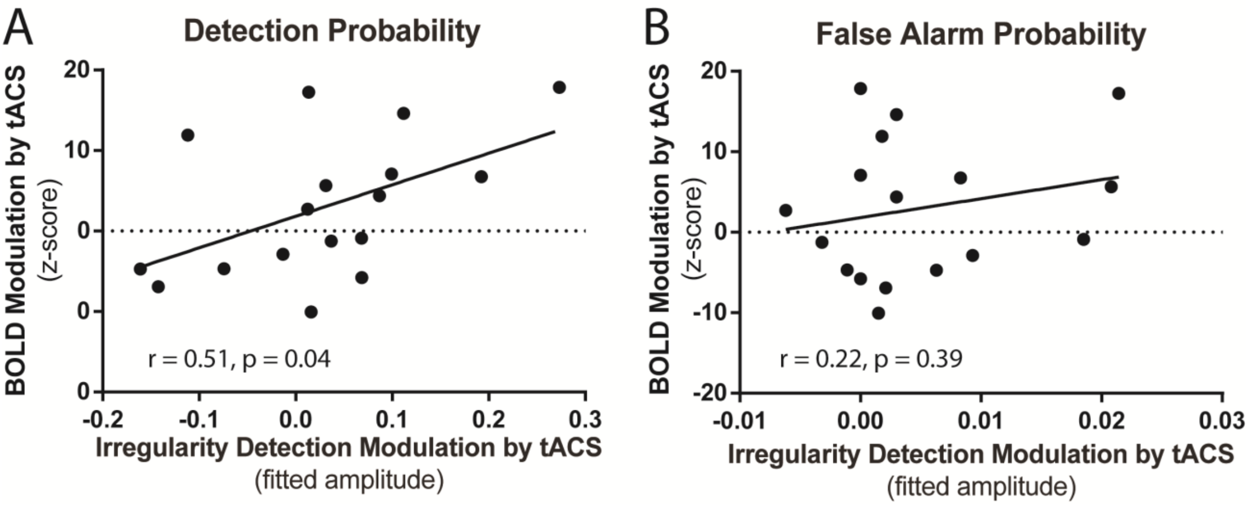

<u>Figure S4</u>. Supplemental correlations between neural & behavioural results. Related to Figure 4. Correlation between modulations of BOLD response and behaviour by the phase relation between tACS and speech in the stimulation/intelligible condition. Shown are results for detection probability (**A**) and false alarm probability (**B**) as performance measures.

<u>Supplemental References</u>

S1. Antal, A., Polania, R., Schmidt-Samoa, C., Dechent, P., and Paulus, W. (2011). Transcranial direct current stimulation over the primary motor cortex during fMRI. NeuroImage *55*, 590–596.

S2. Stagg, C.J., and Nitsche, M.A. (2011). Physiological basis of transcranial direct current stimulation. Neurosci. Rev. J. Bringing Neurobiol. Neurol. Psychiatry *17*, 37–53.
